# RORγt^+^ cells selectively express redundant cation channels linked to the Golgi apparatus

**DOI:** 10.1038/srep23682

**Published:** 2016-03-24

**Authors:** Lucile Drujont, Aurélie Lemoine, Aurélie Moreau, Géraldine Bienvenu, Mélanie Lancien, Thierry Cens, Flora Guillot, Gaëlle Bériou, Laurence Bouchet-Delbos, Hans Jörg Fehling, Elise Chiffoleau, Arnaud B. Nicot, Pierre Charnet, Jérôme C. Martin, Régis Josien, Maria Cristina Cuturi, Cédric Louvet

**Affiliations:** 1 INSERM UMR 1064, Center for Research in Transplantation and Immunology; Université de Nantes; CHU Nantes, Institut de Transplantation Urologie Néphrologie (ITUN); 44093 Nantes, France; 2 CNRS UMR 5237, CRBM, 34293 Montpellier, France; 3 Institute of Immunology, University Clinics Ulm, 8901 Ulm, Germany; 4 Faculté de Médecine, Université de Nantes, 44093 Nantes, France; 5 Laboratoire d’Immunologie, CHU Nantes, 44093 Nantes, France

## Abstract

Retinoid-related orphan receptor gamma t (RORγt) is a master transcription factor central to type 17 immunity involving cells such as T helper 17, group 3 innate lymphoid cells or IL-17-producing γδ T cells. Here we show that the intracellular ion channel TMEM176B and its homologue TMEM176A are strongly expressed in these RORγt^+^ cells. We demonstrate that TMEM176A and B exhibit a similar cation channel activity and mainly colocalise in close proximity to the *trans*-Golgi network. Strikingly, in the mouse, the loss of *Tmem176b* is systematically associated with a strong upregulation of *Tmem176a*. While *Tmem176b* single-deficiency has no effect on the course of experimental autoimmune encephalomyelitis, T cell or DSS-induced colitis, it significantly reduces imiquimod-induced psoriasis-like skin inflammation. These findings shed light on a potentially novel specific process linked to post-Golgi trafficking for modulating the function of RORγt^+^ cells and indicate that both homologues should be simultaneously targeted to clearly elucidate the role of this intracellular ion flow.

CD4^+^ Th17 cells were definitely recognised as a distinct Th subset, along with Th1 and Th2, a decade ago owing to the identification of RORγt as their master transcription factor^1^. While Th1 and Th2 cells are required for the control of intracellular pathogens or extracellular worms respectively, Th17 cells appear essential for proper defence against extracellular bacteria and fungi^2^. Moreover, it is now established that deregulated IL-17 secretion by Th17 cells also contributes to the development of several immune-mediated inflammatory diseases (IMIDs)^3^, and a number of clinical trials aiming at evaluating the therapeutic value of IL-17 or IL-17R blockade have been conducted that led to both impressive and disappointing results, depending on the disease targeted^4^,^5^. Interestingly, RORγt function as a master regulator of transcription is not restricted to Th17 cells but also extends to group 3 innate lymphoid cells (ILC3s), which are regarded as their innate counterparts^6^. Additionally, RORγt^+^ expression is also detected in IL-17-producing γδ T cells that emerge as important players in inflammatory diseases as well^7^,^8^,^9^,^10^. Unveiling novel and specific aspects of RORγt^+^ lymphocytes beyond their cytokine production is thus important to better understand their actions during physiological and/or deregulated immune responses.

*Tmem176b*, initially named *Torid* (*tolerance-related and induced*), encodes a four-span transmembrane protein that we previously showed to be expressed in immature myeloid cells, including conventional dendritic cells (cDCs)^11^,^12^. More recently, we demonstrated that TMEM176B exerts major functions in the cross-presentation of antigens by tolerogenic DCs through acting as a non-selective cation channel that finely regulates the phagosomal pH^13^. Importantly, *Tmem176b* has a co-regulated homologue, namely *Tmem176a*, located within the same genomic locus and likely resulting from a recent duplication^11^,^12^,^14^. It is important to note that the co-expression of these two structurally similar genes is not restricted to myeloid cells but extends to other cell types including non-hematopoietic cells^15^,^16^,^17^,^18^, thus strongly suggesting other intracellular functions beyond antigen cross-presentation.

A recent study by Littman and colleagues^19^ revealed that, along with *Il17a*, *Il17f, Il23r*, *Ccl20*, *Il1r1* or *Ltb4r1*, *Tmem176a* and *b* are part of the highly restricted group of 11 genes whose expression is directly dependent on RORγt in Th17 cells. Concordant with this, a significant upregulation of both homologues was detected in whole-blood samples of patients with multiple sclerosis^20^, an IMID in which the pathogenic role of type 17 immunity is strongly suspected^21^,^22^,^23^,^24^. More recently, as part of the Immunological Genome Project, Colonna and colleagues highlighted several ILC-specific genes including *Tmem176a* and *b*, whose expression was remarkably higher in ILC3 subsets than in group 1 and 2 ILCs^25^. These findings thus logically raise the question whether *Tmem176a* and *b* play a role in type 17 immunity-related RORγt^+^ lymphocytes, including Th17 cells and ILC3s, which is yet to be unveiled.

In the present study, we have characterised *Tmem176a* and *b* expression in RORγt^+^ lymphocytes at transcriptional and protein levels and present evidence that both genes exert a redundant ion channel function related to a colocalisation in close proximity to the Golgi apparatus.

## Results

### *Tmem176a* and *Tmem176b* are over-expressed in Th17 cells

**Figure 1:**
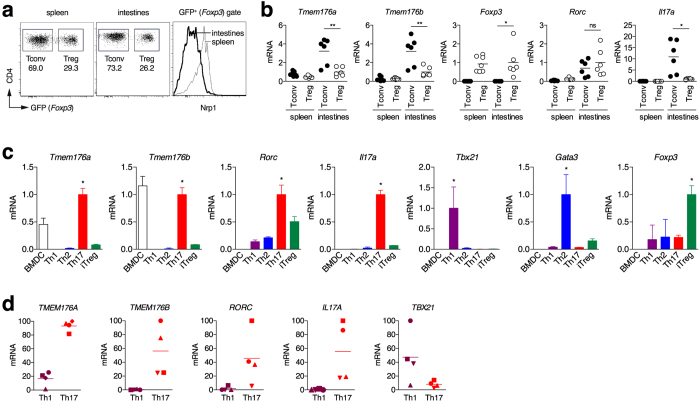
*Tmem176a* and *b* mRNA expression in mouse and human T cells. (**a**) Conventional GFP^−^ (Foxp3^−^ Tconv) or regulatory GFP^+^ (Foxp3^+^ Treg) CD4^+^ T cells were FACS-sorted from the spleen or intestinal lamina propria (small intestine and colon) of *Foxp3*^*EGFP*^ mice. As expected, the population of Nrp1^−/low^ “adaptive” peripherally Tregs is dominant in the intestines. Conversely, Nrp1^+^ “natural” thymically derived Tregs represent the major population of Tregs in spleen. (**b**) Expression of indicated genes was assessed by quantitative RT-PCR. Each dot represents an individual mouse (n = 6–7 in each group). Statistically significant differences between intestinal Tconv and Treg are indicated: *p < 0.05, **p < 0.01. (**c**) Mouse naive CD4^+^ T cells were stimulated with anti-CD3 and anti-CD28 under Th1, Th2, Th17 or iTreg polarising conditions for 3 days. Immature bone marrow-derived DCs (BMDC) were generated with GM-CSF. Expression of indicated genes was assessed by quantitative RT-PCR. Data show triplicates (mean ± SD) for each condition and are representative of three independent experiments. *indicates a statistically significant difference of the indicated population compared to all the other Th cells. (**d**) Human naive CD4^+^ T cells were stimulated with anti-CD3 and anti-CD28 under Th1 or Th17 polarising conditions for 7 days. Expression of indicated genes was assessed by quantitative RT-PCR by comparing Th1 and Th17 obtained from 4 independent healthy volunteers. Each donor is coded by a specific symbol.

We previously reported very low expression of *Tmem176a* and *b* mRNA in naive or anti-CD3/CD28 stimulated T cells^11^,^12^. However, whether these genes are upregulated in terminally differentiated T cell subsets has not been investigated. To this end, we took advantage of *Foxp3*^*EGFP*^ reporter mice that specifically express GFP under the control of the *Foxp3* promoter to purify Foxp3^+^ (GFP^+^) Tregs along with Foxp3^−^ (GFP^−^) conventional T cells (Tconv) from the spleen in which most of the Tregs are thymically-derived (Nrp1^+^), and the gut lamina propria in which specific environmental factors strongly drives the differentiation of peripherally derived Tregs (Nrp1^lo^) specific for food and commensal antigens (Fig. 1a). Concordant with published microarray data suggesting a preferential expression of *Tmem176a* and *b* in peripherally derived Tregs^26^, we detected significantly higher mRNA levels of both homologues in intestinal Tregs as compared to splenic Tregs. However, the highest levels of expression were actually found specifically in intestinal Tconv cells (Fig. 1b), pointing to Th17 cells, the major T helper cell subset in the gut^27^, as another important population expressing *Tmem176a* and *b*.

The expression of *Tmem176a* and *b* was therefore assessed in *in vitro* polarised Th cell subsets. As shown in Fig. 1c, expression of both genes was markedly induced in Th17 cells and, to a much lower extent, in induced Tregs (iTregs) but not in Th1 or Th2 cells. Within the lymphoid lineage, this expression profile mirrors the ones of *Rorc* or *Il17a* and is consistent with the findings reported by Ciofani *et al*.^19^ showing that *Tmem176a* and *b* are direct targets of RORγt in Th17 cells. Importantly, these results hold true in human T cells as high levels of both *TMEM176A* and *B* human orthologues were also found in *in vitro* polarised Th17 cells (Fig. 1d).

### *Tmem176a* and *Tmem176b* are strongly expressed in ILC3s

**Figure 2:**
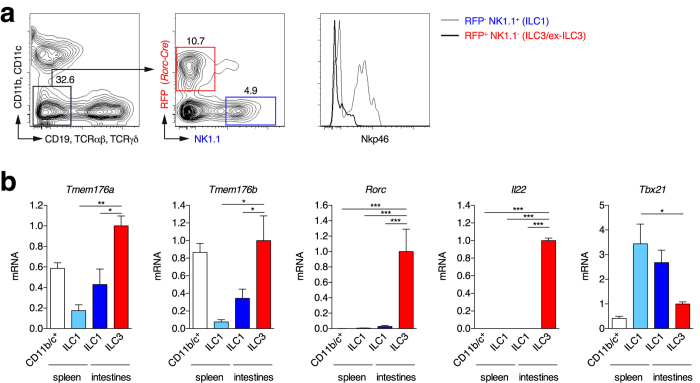
*Tmem176a* and *b* mRNA expression in intestinal ILC3s. (**a**) Lymphocytes from intestinal lamina propria of RORγt-fate map mice (*Rorc*(*γt*)-*Cre*^TG^ × *Rosa26-tdRFP*) were isolated. Lineage-negative (CD11b^−^CD11c^−^CD19^−^TCRαβ^−^TCRγδ^−^) RFP^+^NK1.1^−^ ILC3-enriched and RFP^−^NK1.1^+^ ILC1s were FACS-sorted. In parallel, CD11b/c^+^ and lineage-negative NK1.1^+^ conventional NK/ILC1s were FACS-sorted from the spleen. (**b**) Expression of indicated genes (mean ± SD) was assessed by quantitative RT-PCR in each population isolated from 3–4 independent mice. Statistically significant differences between ILC3s and the other populations are indicated: *p < 0.05, **p < 0.01, ***p < 0.001.

Similarly to Th17 cells, ILC3s require RORγt for their development^6^, thus suggesting that high levels of *Tmem176a* and *b* expression should also be detected in these cells. To test for this hypothesis, we took advantage of a RORγt-fate map mouse (*Rorc* (*γt*)*-Cre*^TG^ × *Rosa26-tdRFP*) in which cells expressing, or having previously expressed, RORγt are permanently marked by the tandem-dimer red fluorescent protein (RFP) and can be easily identified (Fig. 2a). We isolated lineage-negative (lin^−^) RFP^+^ ILC3s and ex-ILC3s from the gut lamina propria^28^, and compared them to spleen and gut lin^−^ RFP^−^ NK1.1^+^ ILC1s (including NK cells) for *Tmem176a* and *b* gene expression. Concordant with our hypothesis, the highest expression levels were indeed detected in the lin^−^ RFP^+^ population (Fig. 2b). In fact, *Tmem176a* and *b* mRNA expression respectively reached or was higher than the levels found in CD11b/c^+^ myeloid cells that were previously considered as the strongest expressors of these two homologues among hematopoietic cells^11^,^12^. Thus, *Tmem176a* and *b* expression appears preferentially overexpressed in RORγt^+^ lymphocytes, at least in Th17 cells and ILC3s.

### *Tmem176a* and *Tmem176b* share a similar cation channel function

**Figure 3:**
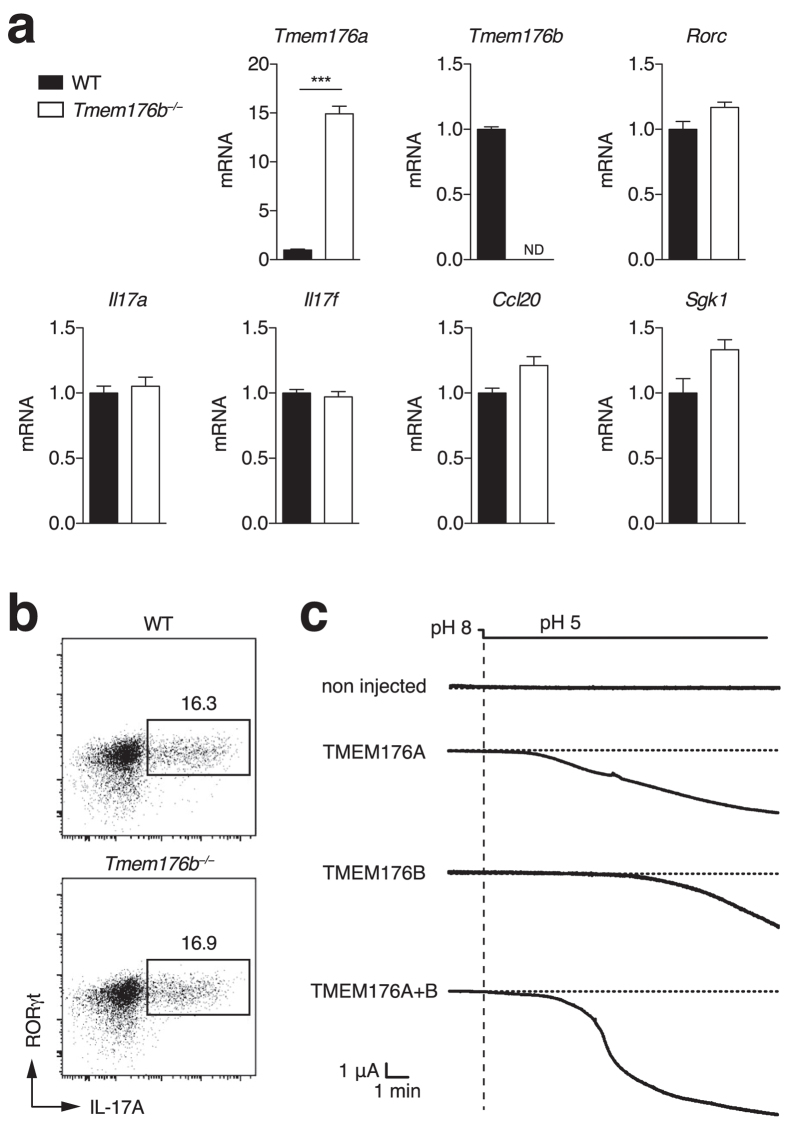
Expression compensation and ion channel activity of *Tmem176a* and *b*. (**a**) Naive CD4^+^ T cells from WT or *Tmem176b*^*−/−*^ mice were stimulated with anti-CD3 and anti-CD28 under Th17 polarising conditions for 3 days. Expression of indicated genes was assessed by quantitative RT-PCR. Data show triplicates (mean ± SD) for each condition and are representative of five independent experiments. **p < 0.01. ND: Not detected. (**b**) Intracellular RORγt and IL-17A expression was assayed by FACS after PMA/ionomycin restimulation. (**c**) *Xenopus* oocytes were injected with *Tmem176a* or/and *Tmem176b* mRNA and currents were recorded in voltage-clamp 2–4 days later. Translocation of TMEM176A and TMEM176B to the plasma membrane was induced by a 30-min treatment with PMA. The currents were quantified 5–15 min after holding the extracellular pH at 5. Representative recordings are shown.

*Tmem176a* and *b* genes are located within the same genomic locus in opposite direction (Fig. S1a), likely arising from the duplication of a common ancestral gene^14^, and are tightly co-regulated in various tissues^11^,^12^. Strikingly, we found that *Tmem176a* expression in Th17 cells was further increased in *Tmem176b*^*−/−*^ compared to WT cells (mean fold change: 12.7 ± 3.5 (SD) from 5 independent experiments) whereas the expression of other Th17-specific genes such as *Rorc*, *Il17a*, *Il17f*, *Ccl20* or *Sgk1*^29^,^30^ were not altered (Fig. 3a). Consistently, IL-17A production revealed by intracellular FACS analysis remained unaltered (Fig. 3b). We^12^ and others^31^ have shown that TMEM176A and B are structurally similar transmembrane proteins that could physically interact. However, given that both homologues are also likely to function independently, *Tmem176a* upregulation might represent a physiological mechanism to compensate for the loss of *Tmem176b* and thus preserve, at least partially, their redundant and therefore presumably important ion channel activity.

We recently demonstrated that *Tmem176b* encodes a non selective monovalent cation channel activated by acidification^13^. Protein sequence comparison across various mammalian species revealed that the most striking amino-acid conservations are concentrated within the three first transmembrane domains (Fig. S1b,c), strongly suggesting that both homologues could exert the same ion channel function. To test for this hypothesis, we expressed TMEM176A or TMEM176B proteins in *Xenopus* oocytes and recorded the electric activity under whole-cell patch clamp after PMA treatment in order to allow surface expression^32^. As shown in Fig. 3c, TMEM176A and B exhibited a similar ability to induce an inward current activated by acidification of the extracellular solution to pH 5, while concomitant expression resulted in the development of an even more intense current.

Taken together, these data show that both TMEM176A and B are cation channels, and suggest that each molecule has the potential to compensate for each other, either in terms of expression or function.

### TMEM176B intracellular localisation is strongly associated with the Golgi apparatus

Having established the ion channel redundancy of TMEM176A and B, we next sought to determine their intracellular localisation in Th17 cells. We previously showed that, in DCs, TMEM176B is expressed in the endophagosomal membranes where its cationic conductance promotes V-ATPase activity and vesicular acidification^13^, a process referred to as counterion conductance. However, the localisation of TMEM176B in DCs is not restricted to the endophagosomes as we consistently observed TMEM176B expression in other intracellular vesicular compartments, notably in the perinuclear area^11^,^13^.

**Figure 4:**
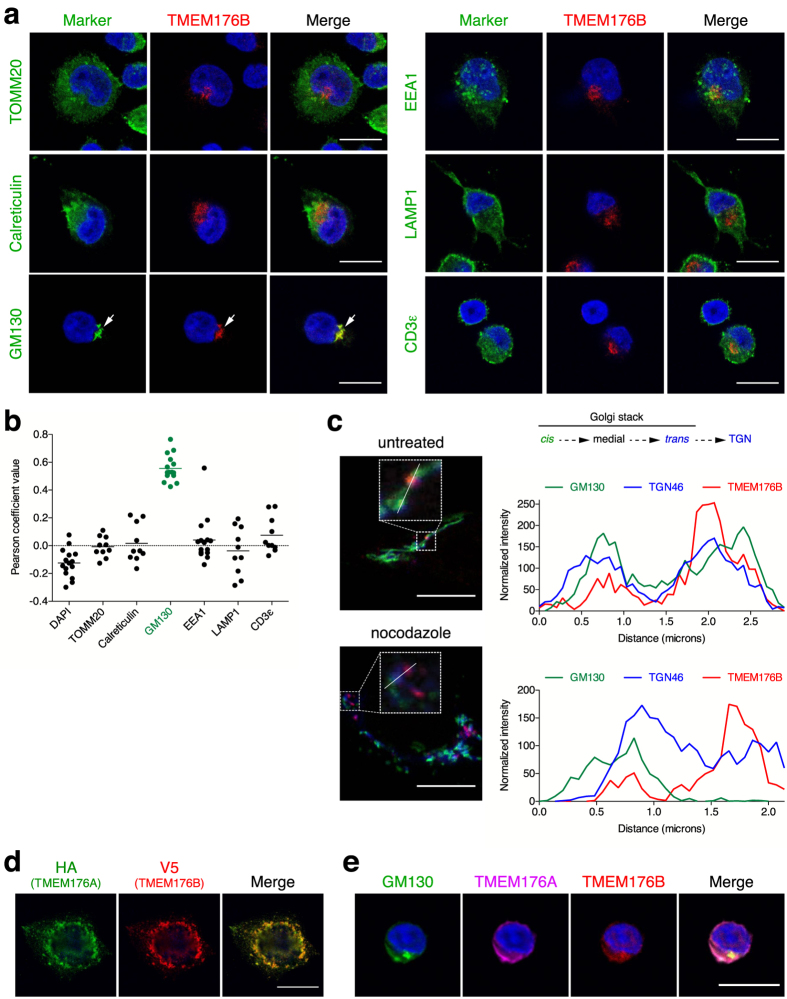
Intracellular localisation of TMEM176A and TMEM176B. (**a**) Human Th17 polarised cells from naive CD4^+^ T cells were coated on microscopy slides, fixed, permeabilised and co-stained for TMEM176B (red) and the indicated markers (green). DAPI was used for nuclear staining (blue). Arrows indicate TMEM176B colocalisation with the *cis*-Golgi protein GM130. Bar, 10 μm. (**b**) Pearson’s correlation coefficients of TMEM176B and the indicated markers (n = 10–15 in each group). (**c**) HeLa cells were treated or not with nocodazole for 4 hr and subsequently fixed, permeabilised and co-stained for TMEM176B (red), GM130 (*cis*-Golgi, green) and TGN46 (*trans*-Golgi and *trans*-Golgi network (TGN), blue). Bar, 10 μm. Insets represent higher magnifications of regions of interest. Linescans show fluorescence intensity along the lines overlaying the images. (**d**) HeLa cells were co-transfected with plasmids encoding TMEM176A-HA and TMEM176B-V5 fusion proteins and subsequently fixed, permeabilised and co-stained with HA (green) and V5 (red) monoclonal antibodies. DAPI was used for nuclear staining (blue). (**e**) Human Th17 polarised cells (as in A) were co-stained for GM130 (green), TMEM176A (purple) and TMEM176B (red). DAPI was used for nuclear staining (blue). Bar, 10 μm.

To determine the precise localisation of TMEM176B, we identified a polyclonal anti-human TMEM176B antibody whose specificity for indirect immunofluorescence application was checked on transiently transfected cells with an expression plasmid. Staining of *in vitro*-polarised human Th17 cells showed a predominant accumulation in a compact juxtanuclear compartment (Fig. 4a) while Th1 cells exhibited almost no staining (Fig. S2). This pattern was distinct from those observed with antibodies against the mitochondria (TOMM20), the endoplasmic reticulum (ER, calreticulin), the early endosomes (EEA1), the lysosomes (CD107a) or the T-cell receptor subunit CD3ε. In contrast, TMEM176B expression was closely apposed to GM130, a Golgi-resident protein, an observation confirmed by colocalisation measurement of several cells (Fig. 4b). Importantly, we found a similar pattern of expression in human monocyte-derived DCs (Fig. S3) as well as in HeLa cells (Fig. S4), strongly suggesting that TMEM176B association with the Golgi is a universal feature found in different types of cells. Of note, TMEM176B did not colocalise with autophagosomes in HeLa cells (Fig. S4).

The Golgi apparatus consists of a collection of stack of cisternae and associated vesicles where proteins and lipids from the ER enter at its *cis* face and exit at its *trans* face. In mammal cells, several stacks concentrate to form a compact Golgi ribbon that precludes precise discrimination of distinct regions. To further define the localisation of TMEM176B in relation with the Golgi, we treated HeLa cells with the microtubule-disrupting agent nocodazole that induces spatial separation of the Golgi stacks^33^. We combined TMEM176B analysis with GM130 (*cis*-Golgi) and TGN46 that marks the *trans*-Golgi face and the *trans*-Golgi network (TGN). Whereas untreated cells showed an expected dense and overlapping expression profile of the three proteins, nocodazole treatment allowed fragmentation of the Golgi apparatus and identification of individual stacks in which *cis* (GM130) to *trans* (TGN46) polarisation was discernable (Fig. 4c). Interestingly, TMEM176B expression was consistently more associated with TGN46 but also appeared clearly beyond this marker. Thus, TMEM176B is probably not a Golgi-resident protein but concentrates in vesicles in close proximity to the TGN from where it could emanate to reach (or be recycled from) the endosomal system, a finding which is coherent with TMEM176B detection in the phagosomal membrane in DCs^13^,^34^.

### TMEM176A and B are colocalised

We next sought to determine the intracellular localisation of TMEM176A relative to TMEM176B. Co-transfection of HeLa cells with plasmids encoding epitope-tagged fusion proteins TMEM176A-HA and TMEM176B-V5 showed a strong colocalisation of these two proteins in the perinuclear region reminiscent of the Golgi apparatus but also in punctate, vesicle-like structures dispersed throughout the cytoplasm (Fig. 4d). Of note, this latter pattern of expression revealed by transfection and clearly outside from the Golgi was also observed in untransfected Hela cells, Th17 cells and DCs by the TMEM176B-specific antibody, although to a lesser extent probably reflecting the detection limit of endogenous expression. In contrast to TMEM176B, we did not succeed in identifying a commercial antibody showing reproducible immunofluorescence specificity to human TMEM176A. We thus generated a rat polyclonal antibody directed against TMEM176A that could be combined with the rabbit antibody against TMEM176B and for which we checked the specificity on cells transiently expressing the fusion protein TMEM176A-HA. As shown in Fig. 4e, human Th17 cells displayed a strong colocalisation of TMEM176A and B along with GM130.

All together, these results show that TMEM176A and B are colocalised in intracellular vesicular structures that concentrate mainly in close association with the *trans* face of the Golgi apparatus, and more precisely the TGN.

### Impact of *Tmem176b* deficiency in mouse models of IMIDs

**Figure 5:**
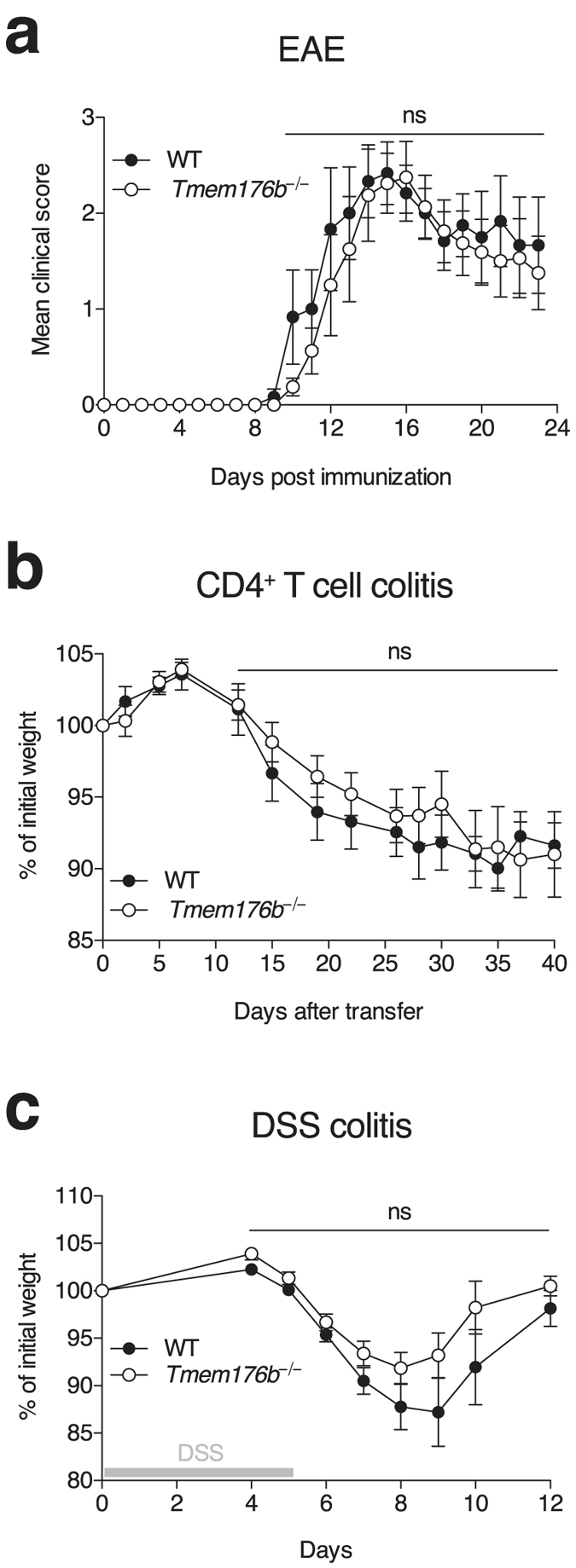
*Tmem176b* single-deficient mouse susceptibility to the development of EAE, chronic and atute colitis. (**a**) EAE was induced in WT (n = 6) and *Tmem176b*^*−/−*^ (n = 8) mice by immunisation (s.c.) with MOG peptide in CFA. Clinical course of disease is shown. (**b**) Chronic colitis was induced in *Rag1*^*−/−*^ mice (n = 11–12 in each group) by adoptive transfer (i.v.) of FACS-sorted CD4^+^CD45RB^hi^ T cells from WT or *Tmem176b*^*−/−*^ mice. Data are presented as percent of initial weight. (**c**) Acute colitis was induced in WT (n = 6) and *Tmem176b*^*−/−*^ (n = 8) mice with 3% DSS in drinking water for 5 consecutive days. Data are presented as percent of initial weight.

High expression of *Tmem176a* and *b* in RORγt^+^ cells suggests that these homologues play a role in immune disorders involving protective or pathogenic actions of these cells. Given that mice deficient for both genes or for *Tmem176a* alone were not available, we chose to analyse the *Tmem176b* single-KO mice that we previously described^13^. We first examined the susceptibility of these mice to the EAE model induced by the MOG_35–55_ peptide, and which allowed the characterisation of RORγt as the master transcription factor for Th17 cells^1^. *Tmem176b* deficiency did not alter the susceptibility of mice to the disease, as we did not observe any significant difference between WT and *Tmem176b*^*−/−*^ animals in terms of onset or severity of the disease (Fig. 5a). Second, we specifically assessed the role of *Tmem176b* in CD4^+^ T cells by using the transfer model of colitis, in which the intrinsic expression of RORγt in CD4^+^CD25^−^CD45RB^hi^ T cells is known to be required to induce a severe colitis after injection into *Rag1*^−/−^ recipients^35^. However, as shown in Fig. 5b, the absence of *Tmem176b* in transferred T cells did not alter the course of the disease. Next, to more specifically address the role of *Tmem176b* in innate cells, we moved to the dextran sulfate sodium (DSS) model of acute colitis. In this model, DSS rapidly induces epithelial damage leading to bacterial translocation and subsequent gut inflammation^36^,^37^. Previous work has demonstrated the crucial role of ILC3-derived IL-22 to restore epithelial barrier integrity and thus recovery of treated mice^38^,^39^. Again, in this model, *Tmem176b*^*−/−*^ mice exhibited no difference with WT animals regarding colitis severity or duration (Fig. 5c).

**Figure 6:**
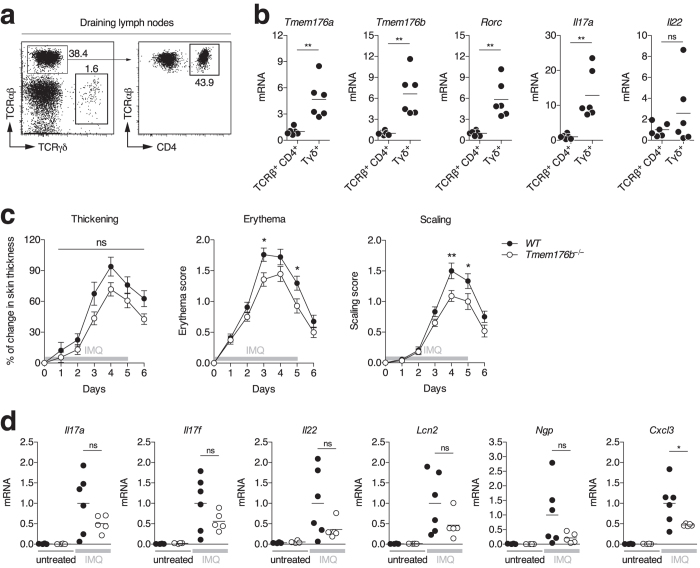
*Tmem176b* single-deficient mouse susceptibility to the development of psoriasis-like dermatitis. (**a**) Psoriasis-like dermatitis was induced in WT mice by topical application of imiquimod (IMQ) cream on the shaved back skin. At day 4, draining (inguinal) lymph nodes were harvested and TCRγδ^+^ and TCRβ^+^CD4^+^ T cells were FACS-sorted. (**b**) Expression of indicated genes was assessed by quantitative RT-PCR. Each dot represents an individual mouse (n = 6 in each group). Statistically significant differences are indicated: **p < 0.01. (**c**) Psoriasis-like dermatitis was induced in WT (n = 27) and *Tmem176b*^*−/−*^ (n = 28) mice by topical application of imiquimod (IMQ) cream on the shaved back skin during 5 consecutive days. Mice were weighed and scored daily on a scale from 0 to 4 for skin thickening, erythema and scaling. *p < 0.05, ***p < 0.01. (**d**) Expression of indicated genes was assessed by quantitative RT-PCR in the skin of untreated or IMQ-treated WT and *Tmem176b*^*−/−*^ mice (n = 4–6 in each group). Statistically significant differences between IMQ-treated WT and *Tmem176b*^*−/−*^ mice are indicated: *p < 0.05.

Lastly, we examined the model of psoriasis-like skin inflammation induced by topical application of Aldara cream (imiquimod, IMQ) where RORγt^+^ γδ T cells and ILC3s, but not αβ T cells, are the primary source of pathogenic IL-17A, IL-17F and IL-22^8^,^9^,^40^. Importantly, γδ T cells isolated from the draining lymph nodes of treated mice (Fig. 6a) exhibited higher expression of *Tmem176a* and *b* than CD4^+^ T cells, similarly to *Rorc* or *Il17a* (Fig. 6b). Interestingly, although thickening was similar, *Tmem176b*^*−/−*^ mice exhibited significantly reduced reddening and scaling of the skin compared to WT mice (Fig. 6c). Furthermore, *Il17a*, *Il17f* and *Il22* as well as induced genes such as *Lcn2* (*lipocalin-2*), *Ngp* (*neutrophilic granule protein*) or *Cxcl3* mRNA levels of expression were decreased in the skin of treated *Tmem176b*^*−/−*^ compared to WT mice but only with *Cxcl3* reaching statistical significance (Fig. 6d).

Thus, *Tmem176b* single-deficiency can be associated with a significant although limited reduction of RORγt-dependent pathological inflammation that reinforces the possibility of compensatory mechanisms mediated by its homologue *Tmem176a*.

## Discussion

The discovery of Th17 cells as a distinct differentiation of T cell has sparked an intense research on their role in host defence and IMIDs. RORγt^+^ lymphocytes comprise other cells than T cells and notably include ILC3s, the innate counterpart of Th17. Much attention has been given to these cells through the prism of key cytokines they produce, namely IL-17A, IL-17F, IL-22 or GM-CSF (CSF2), leading to mucosal defence and repair. In contrast to plasma membrane ion channels^41^, little is known about intracellular ion flows, especially if these channels are involved in specific immune cells. The high expression of TMEM176A and B cation channels in RORγt^+^ cells is intriguing and could represent a novel therapeutic entry point for treating IMIDs.

Here we demonstrate that the homologues *Tmem176a* and *b* are strongly expressed in Th17 as compared to other CD4^+^ T cells subsets both in mouse and human. These results extend the work of Ciofani *et al*. which revealed that, like *Il17a* and *f, Tmem176a* and *b* are direct targets of RORγt in Th17 cells^19^. In line with these results, the expression levels of *Tmem176a* and *b* were found significantly decreased after pharmacological treatment specifically targeting Th17 transcriptional program^42^. We also found that Foxp3^+^ Tregs exhibit a significant expression of *Tmem176a* and *b*, although to a lesser extent than Th17 cells, likely related to the presence of double positive Foxp3^+^RORγt^+^ cells that now emerge as regulators of specific helper T cell responses in the colon^43^,^44^. Interestingly, with regard to Tregs, *Tmem176a* and *b* have recently been shown to be preferentially expressed in amphiregulin-producing IL-10^−^IL-18R^+^ Tregs that are proficient in tissue repair^45^.

It is tempting to link the cation channel function of TMEM176A and B with the recent findings of the effect of sodium on Th17 cells^29^,^30^, Tregs^46^ or M2 macrophages^47^. Furthermore, *TMEM176A* and *B* mRNA upregulation in whole blood cells of MS patients^20^ raises the possibility of a causal connection with high dietary salt that has been associated with increased disease activity^48^.

Reasoning that *Tmem176a* and *b* may also be induced in other RORγt^+^ lymphocytes, we found high levels of expression in intestinal ILC3s. Of note, given that RFP^+^ cells from RORγt-fate map mouse contain a substantial proportion of RORγt^−^ ex-ILC3s, this expression was likely underestimated, notably because intestinal lamina propria preparations included the colon which is particularly permissive for RORγt loss in ILC3s^28^. Retrospective analysis of published microarray data comparing lung ILC2 and spleen ILC3s^49^ revealed a clear differential expression of *Tmem176a* and *b* in ILC3s. However, this expression does not appear restricted to ILC3s since we found it substantially increased in ILC1s from the intestines compared to the spleen (conventional NK cells). In fact, Colonna and colleagues have recently shown that *Tmem176a* and *b* expression was also significantly increased in both ILC1s and ILC2s from the small intestine compared to conventional NK^25^. Thus, although *Tmem176a* and *b* highest levels of expression were found in ILC3, these genes could also play key roles in other types of ILCs, notably in the intestinal environment where local factors could favour their induction independently of RORγt.

Besides Th17 cells and ILC3s, we also found that IL-17-producing γδ T cells preferentially expressed *Tmem176a* and *b*. In fact, the development of psoriasis-like dermatitis in which these cells are central was partially reduced in *Tmem176b* single-deficient mice, suggesting an important intrinsic role of both homologues in these emerging players of type 17 immunity.

Based on the *in vivo* and *in vitro* data presented here, we propose that TMEM176A and B exert a redundant ion channel function at the interface of the endosomal network and the Golgi apparatus. It is then likely that pronounced phenotypes will be observed only when the two homologues are simultaneously targeted. To this end, we are currently generating a double KO mouse that will represent an invaluable tool to elucidate the role of these cation channels in RORγt^+^ cells or other cells such as selected DC subsets in which they are highly expressed^50^,^51^,^52^. Furthermore, conditional tissue-specific deletion will be instrumental to draw definitive conclusions on the contribution of *Tmem176a* and *b* in virtually any specific cell types.

To date, only one Golgi-resident ion channel (GPHR: Golgi pH regulator, *Gpr89*) have been reported to allow a pH gradient from the *cis* to the *trans* face of the Golgi apparatus that is crucial for optimal protein transport or glycosylation^53^. Counterion conductance generated by TMEM176B^13^ and presumably by TMEM176A could be required for further acidification control of organelles linked to the TGN (*trans* Golgi network) such as maturing endophagosomes or secretory vesicles^54^ and thus directly participate in the dynamic of the endocytic and exocytic system which is central for the cells to communicate with their surroundings. According to this hypothesis, we previously showed that non-physiological overexpression of *Tmem176b* in DCs altered their capacity to upregulate MHC II and CD86 molecules at the surface and to secrete IL-12^11^. Taking into consideration the pivotal role of RORγt^+^ cells in tissues that interface with the microbial environment, these cells could foster specific intracellular processes required for their sentinel function and in which TMEM176A and B are involved.

## Methods

### Mice

All mice in this study are on the C57BL/6 background. C57BL/6J mice were purshased from Centre d’élevage Janvier (France). *Foxp3*^*EGFP*^ reporter mice^55^ were provided by Bernard Malissen. BAC transgenic *Rorc*(*γt*)*-Cre*^TG^ mice^56^ (provided by Gérard Eberl) and *Rosa26-tdRFP* reporter mice^57^ (generated by Hans Jörg Fehling and provided by Bernard Malissen) were crossed to obtain a RORγt-fate map mouse. *Tmem176b*^*−/−*^ mice were previously described^13^ and backcrossed for 12 generations onto the C57BL/6J background. *Rag1*^*−/−*^ were obtained from Institut Curie (Paris). All mice were kept under specific pathogen-free conditions and experimental procedures were carried out in strict accordance with the protocols approved by the Commitee on the Ethics of Animal Experiments of Pays de la Loire and authorized by the French Government’s Ministry of Higher Education and Research.

### FACS analysis and cell sorting

Antibodies used in this study for FACS analysis and sorting are listed in Supplementary Table 1. Red blood cells were lysed with ammonium chloride. CD16/32 antibody (BD Biosciences) was used to block the non-specific binding to Fc receptors before all stainings. Dead cells were excluded using DAPI (Invitrogen). For Tconv and Treg isolation from *Foxp3*^*EGFP*^ reporter mice, CD45^+^TCRβ^+^CD4^+^ T cells, respectively GFP^−^ and GFP^+^, were FACS-sorted (BD FACS ARIA^TM^ IIu). Nrp1 staining was added to verify that the proportion of Nrp1^−/low^ “adaptive” pTregs was substantially increased in the intestines compared to the spleen. For ILC3 isolation, CD45^+/low^ lineage-negative (CD11b^−^CD11c^−^CD19^−^TCRαβ^−^TCRγδ^−^) RFP^+^NK1.1^−^ cells were FACS-sorted from the intestines of RORγt-fate map mice (*Rorc*(*γt*)*-Cre*^TG^ × *Rosa26-tdRFP*). Lineage-negative NK1.1^+^ ILC1s were FACS-sorted from spleen or intestines. CD11b^+^CD11c^+^ myeloid cells were FACS-sorted from the spleen. TCRγδ^+^ and TCRβ^+^CD4^+^ T cells were FACS-sorted from inguinal lymph nodes of mice developing psoriasis-like dermatitis. All populations were isolated (>10^5^ cells per mouse) to a purity of >98%.

### Mouse *in vitro* Th polarisation

Spleens were harvested from adult mice and red blood cells were lysed with ammonium chloride. CD4^+^ T cells were enriched using magnetic-activated cell sorting (Miltenyi Biotec) and live (DAPI^−^) CD4^+^CD25^−^CD62L^+^CD44^lo/−^ naive T cells were subsequently FACS-sorted to a purity of >98%. Cells were then cultured during 3 days in 48-well plates (5 × 10^5 ^cells per well) coated with 5 μg/mL anti CD3ε (145-2C11, BD Biosciences) in complete DMEM medium with GlutaMAX (Invitrogen) with 2 μg/mL soluble anti CD28 (37.51, BD Biosciences). Cultures were supplemented as follows. Th1: IL-12 (2 ng/mL, PeproTech) + neutralising anti IL-4 (2 μg/mL, eBioscience); Th2: IL-4 (2 ng/mL, eBioscience) + neutralising anti IFNγ (2 μg/mL, eBioscience); Th17: IL-6 (20 ng/mL, Sigma) + TGF-β (0.3 ng/mL, R&D systems) + neutralising anti IFNγ and anti IL-4; iTreg: TGF-β (5 ng/mL) + neutralising anti IFNγ and anti IL-4. Gene expression in Th1, Th2, Th17 and iTregs was compared to immature bone marrow-derived DCs generated as previously described^13^.

### Human *in vitro* Th polarisation

Peripheral blood samples were collected from healthy donors upon written informed consent and approval by the Institutional Review Board (Etablissement Français du Sang Pays de la Loire, Nantes, France). This study was conducted in accordance with the approved guidelines by the Declaration of Helsinki. Live (DAPI^−^) CD3^+^CD4^+^CD45RO^−^CD45RA^+^ naive T cells were FACS-sorted to a purity of >98% and then cultured during 6 days in 96-well round-bottom plates (50,000 cells per well) coated with 5 μg/mL anti CD3ε (OKT3, prepared in the laboratory) in complete RPMI medium (Invitrogen) with 1 μg/mL soluble anti CD28 (CD28.2, prepared in the laboratory). Cultures were supplemented as follows. Th1: IL-12 (10 ng/mL, R&D systems); Th17: TGF-β (12.5 ng/mL, Peprotech) + IL-1β (5 ng/mL, Peprotech) + IL-6 (25 ng/mL, Peprotech) + IL-23 (25 ng/mL, Peprotech).

### Quantitative RT-PCR

Total RNA from cells was isolated using RNeasy Mini Kit (Qiagen). Skin samples were powderised at low temperature before total RNA isolation using TRIzol^®^ Reagent (Invitrogen, Carlsbad, CA). Reverse transcription was performed using M-MLV Reverse Transcriptase and random primers following manufacturer’s instructions (Invitrogen). Gene expression was assessed with the Fast SYBR Green Master Mix reagent (Applied Biosystems, Foster City, CA). Mouse and human-specific primers used in this study (listed in Supplementary Table 2) were all designed over different exons to prevent amplification of genomic DNA. Real-time PCR was performed using the ViiA 7 Real-Time PCR System (Applied Biosystems). For both mouse and human, gene expression was normalised to glyceraldehyde 3-phosphate dehydrogenase (*Gapdh/GAPDH*) and expressed in arbitrary units using the 2^−ΔΔCt^ method.

### Experimental Autoimmune-Encephalomyelitis (EAE) induced by immunisation with MOG_35–55_ peptide

Mice aged 8–12 weeks were immunised subcutaneously at the base of the tail and lower flanks with 200 μg of MOG_35–55_ peptide (MEVGWYRSPFSRVVHLYRNGK, purity >85%, Genecust Europe, France) emulsified with complete Freund’s adjuvant supplemented with Mycobacterium tuberculosis H37Ra at 6 mg/mL (Difco Laboratories, Detroit, MI, USA). Pertussis toxin (300 ng) was injected intraperitoneally (i.p.) on the day of immunisation and 2 days later. (Calbiochem, Darmstadt, Germany). Mice were scored daily for EAE clinical signs on a scale of 0–5: 0, no disease; 1, complete limp tail; 2, limp tail with unilateral hindlimb paralysis; 3, bilateral hindlimb paralysis; 4, bilateral hindlimb paralysis and forelimb weakness (end point). The observer was blinded to the genotype during the scoring.

### Chronic colitis induced by CD4^+^ T cell adoptive transfer

Naive CD4^+^CD25^−^CD45RB^hi^ T cells from the spleen were FACS-sorted to a purity of >98% and 5 × 10^5^ cells were injected intravenously into C57BL/6 *Rag1*^*−/−*^ recipients aged 8–12 weeks. Mice were monitored and weighed daily.

### Acute colitis induced by dextran sulfate sodium (DSS)

Mice aged 8–12 weeks were given 3% DSS (36,000–50,000 MW, MP Biomedical, Santa Ana, CA) in drinking water ad libitum as indicated for 5 days followed by a recovery period without DSS. Mice were monitored and weighed daily.

### Psoriasis-like skin inflammation induced by imiquimod (IMQ) cream

Backs of mice mice aged 8–12 weeks were shaved with an electric clipper and then treated with depilatory cream (Veet) to remove hair. Two days later, mice received a daily topical dose of 62,5 mg of commercially available IMQ cream 5% (Aldara, 3M Pharmaceuticals) on the shaved back for 5 consecutive days, as previously described^58^. Mice were scored daily for skin inflammation. Dorsal skin thickening was determined by measuring double-skinfold thickness using a digital micrometer (Mitutoyo). Erythema and scaling were scored independently on a scale from 0 to 4: 0 = none, 1 = slight, 2 = moderate, 3 = marked, 4 = severe. For quantitative RT-PCR analysis, back skins were harvested from mice treated with IMQ at day 4 or from untreated mice.

### Confocal microscopy analysis

Antibodies used in this study for confocal microscopy analysis are listed in supplementary Table 3. Th17 cells were were plated on poly-L-lysine-coated slides (Thermo Scientific) for 1, 5 hr in complete medium at 37 °C. The cells were then washed with PBS and fixed in 4% paraformaldehyde, 4% sucrose for 15 min at RT followed by 3 washes with PBS and permeabilisation in 0.05% Triton-X100 for 15 min ar RT. Stainings were then directly performed with primary antibodies (diluted in PBS) for 2 h at RT followed by 3 washes with PBS (5 min each at RT) and stainings with adapted secondary antibodies conjugated with fluorochromes (or biotin) for 30 min at RT. A step with fluorochrome-conjugated streptavidin was added when necessary. After 3 washes with PBS (5 min each at RT), DAPI (diluted in PBS) was incubated 10 min at RT. Slides were mounted with ProLong Gold antifade (Invitrogen). Images were obtained with A1 R Si Confocal microscope (Nikon, Champigny sur Marne, France) and analysed with Fiji software. Pearson’s correlation coefficients were calculated with Volocity software (PerkinElmer).

HeLa were seeded in 8-well μ-Slide (ibidi) and immunostaining was achieved as described above for Th17. HeLa cells were treated with 10 μM nocodazole (Sigma) for 4 hr prior fixation to disrupt the juxtanuclear Golgi ribbon. Linescan data were generated with Fiji software and plotted using Graphpad Prism software (La Jolla, CA). HeLa cells were transfected (Lipofectamine 2000, Invitrogen) with expression plasmids (pCI-neo, Promega) in which we cloned human *TMEM176A* (NM_018487.2) (codon optimisation was performed on a 5′ region of *TMEM176A* sequence to decreased G/C content) or *TMEM176B* (NM_014020.3) cDNA fused (C-term) to HA (YPYDVPDYA) or V5 (GKPIPNPLLGLDST) epitopes, respectively. After 48 hr, the cells were fixed, permeablized and co-stained with monoclonal antibodies against HA or V5 followed by secondary antibody staining. HA and V5 specificity was verified on cells separately transfected with TMEM176A-HA or TMEM176B-V5-encoding plasmids.

### Statistical analysis

All statistical analyses were performed using Graphpad Prism software (La Jolla, CA) with two-tailed unpaired Student’s t test or one-way ANOVA followed by Tukey’s post hoc test. P values < 0.05 were considered significant.

## Additional Information

**How to cite this article**: Drujont, L. *et al*. RORγt^+^ cells selectively express redundant cation channels linked to the Golgi apparatus. *Sci. Rep*. **6**, 23682; doi: 10.1038/srep23682 (2016).

## Supplementary Material

Supplementary Information
