# Supplementary material for: RORγt+ cells selectively express redundant cation channels linked to the Golgi apparatus
Source: Sci Rep. 2016 Mar 24;6:23682. doi: 10.1038/srep23682 (PMC4806298; doi:10.1038/srep23682)
Supplement: Supplementary Information [file srep23682-s1.pdf]

Supplementary Informations

RORγt+ cells selectively express redundant cation channels linked to the Golgi apparatus

Lucile Drujont[1], Aurélie Lemoine[1], Aurélie Moreau[1], Géraldine Bienvenu[1], Mélanie Lancien[1], Thierry Cens[2], Flora Guillot[1], Gaëlle Bériou[1], Laurence Bouchet-Delbos[1], Hans Jörg Fehling[3], Elise Chiffoleau[1], Arnaud Nicot[1], Pierre Charnet[2], Jérôme Christophe Martin[1,4,5], Régis Josien[1,4,5], Maria Cristina Cuturi[1,6] and Cédric Louvet[1,6,*]

[1] INSERM UMR 1064, Center for Research in Transplantation and Immunology; Université de Nantes; CHU Nantes, Institut de Transplantation Urologie Néphrologie (ITUN); 44093 Nantes, France.

[2] CNRS UMR 5237, CRBM, 34293 Montpellier, France.

[3] Institute of Immunology, University Clinics Ulm, 8901 Ulm, Germany

[4] Faculté de Médecine, Université de Nantes, 44093 Nantes, France.

[5] Laboratoire d'Immunologie, CHU Nantes, 44093 Nantes, France.

[6] Co-senior authors.

[*] Corresponding author.

Supplementary Figure Legends

Figure S1. *Tmem176a/b* genomic locus, amino acid and structural identities of TMEM176A and TMEM176B homologs. (**a**) Organisation of *Tmem176a/b* mouse genomic locus (6 B2.3; 6). For each gene, exons are shown (boxes) with untranslated (light) regions and translated (dark, ATG : start codon). (**b**) TMEM176A and B protein sequences from mouse (NP_079602.4, NP_075543.1), rat (NP_001034097.1, NP_599217.1), dog (XP_532758.1, XP_005629732.1), pig (XP_005657767.1, XP_003484042.1), cattle (NP_991348.1, NP_001092615.1), chimpanzee (XP_009452826.1, XP_003318950.1) and human (NP_060957.2, NP_054739.3) were aligned using Clustal Omega (http://toolkit.tuebingen.mpg.de/clustalw). The four predicted transmembrane domains are indicated (grey). Complete (red) and partial (orange) identities were mainly found in the N-terminal region and within the three first transmembrane domains. (**c**) Graphical representation of the topology of mouse TMEM176A and B proteins using Protter[1] (http://wlab.ethz.ch/protter/start/).

Figure S2. TMEM176B protein expression in Th1 and Th17 cells. Human Th1 or Th17 polarized cells from naive $CD4^+$ T cells were coated on microscopy slides, fixed, permeabilized and stained for TMEM176B (red). DAPI was used for nuclear staining (blue). Bar, 10 µm.

Figure S3. Intracellular localization of TMEM176B in human monocyte-derived DCs. (**a**) Monocytes were isolated from peripheral blood mononuclear cells from healthy volunteers donors (French Blood Service, Nantes, France) and cultured for 7 days in complete RPMI medium

(Invitrogen) supplemented with recombinant human GM-CSF (1000 U/mL) and IL-4 (200 U/mL) (CellGenix, Germany) in 6-well plates. Immature monocyte-derived DCs (Mo-DCs) were then coated on microscopy slides, fixed, permeabilized and co-stained for TMEM176B (red) and the indicated markers (green). DAPI was used for nuclear staining (blue). Arrows indicate TMEM176B colocalization with the *cis*-Golgi protein GM130. Bar, 10 μm. (**b**) Pearson's correlation coefficients of TMEM176B and the indicated markers (n = 10–17 in each group).

Figure S4. Intracellular localization of TMEM176B in HeLa cells. (**a**) HeLa were cultured in 8-well μ-Slide (ibidi) and then fixed, permeabilized and co-stained for TMEM176B (red) and the indicated markers (green). DAPI was used for nuclear staining (blue). Arrows indicate TMEM176B colocalization with the *cis*-Golgi protein GM130. Bar, 10 μm. (**b**) HeLa cells were treated with 1 uM Rapamycin and 20 uM chloroquine (CQ) (Enzo Life Sciences) for 18 hr at 37°C to induce the formation and accumulation of autophagosomes[2] detected by LC3-specific antibody (Nanotools) or Cyto-ID Green dye (Enzo Life Sciences). (**c**) Pearson's correlation coefficients of TMEM176B and the indicated markers (n = 7-10 in each group).

Figure S1

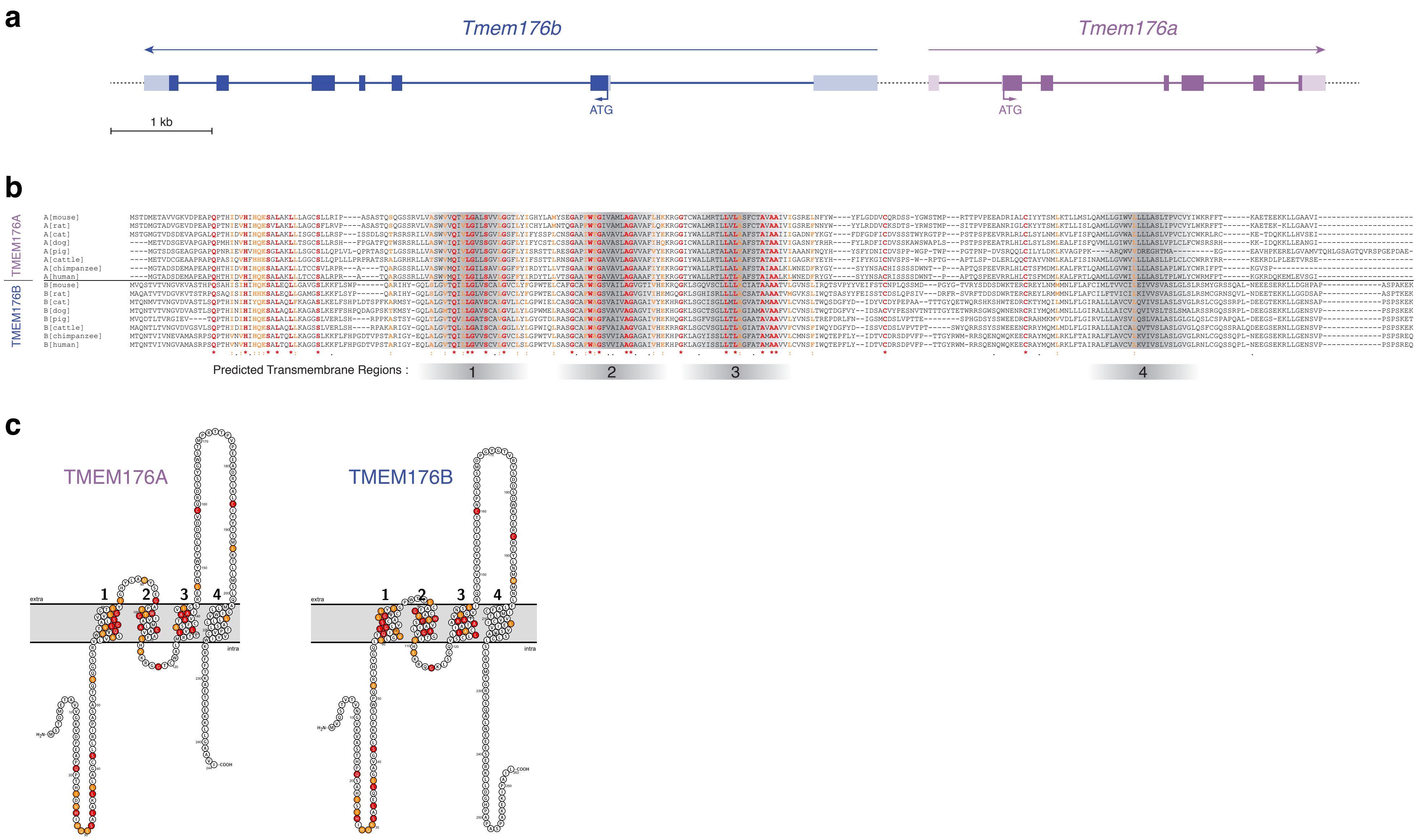
a
Tmem176b
Tmem176a
ATG
ATG
1 kb
b
TMEM176A
TMEM176B
Predicted Transmembrane Regions :
1
2
3
4
c
TMEM176A
TMEM176B
extra
intra
1
2
3
4
H2N-
-COOH

Figure S2

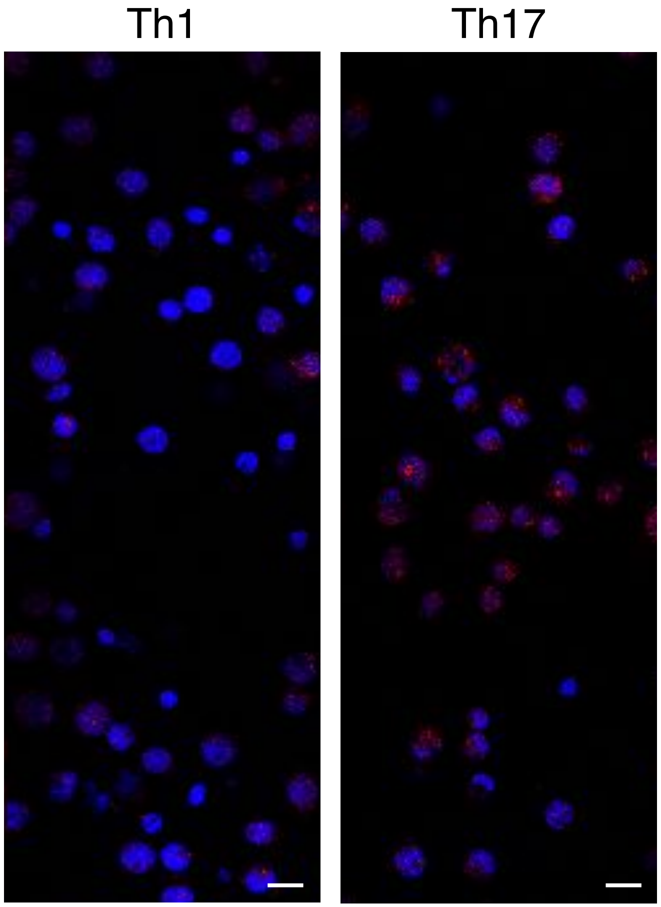

Figure S3

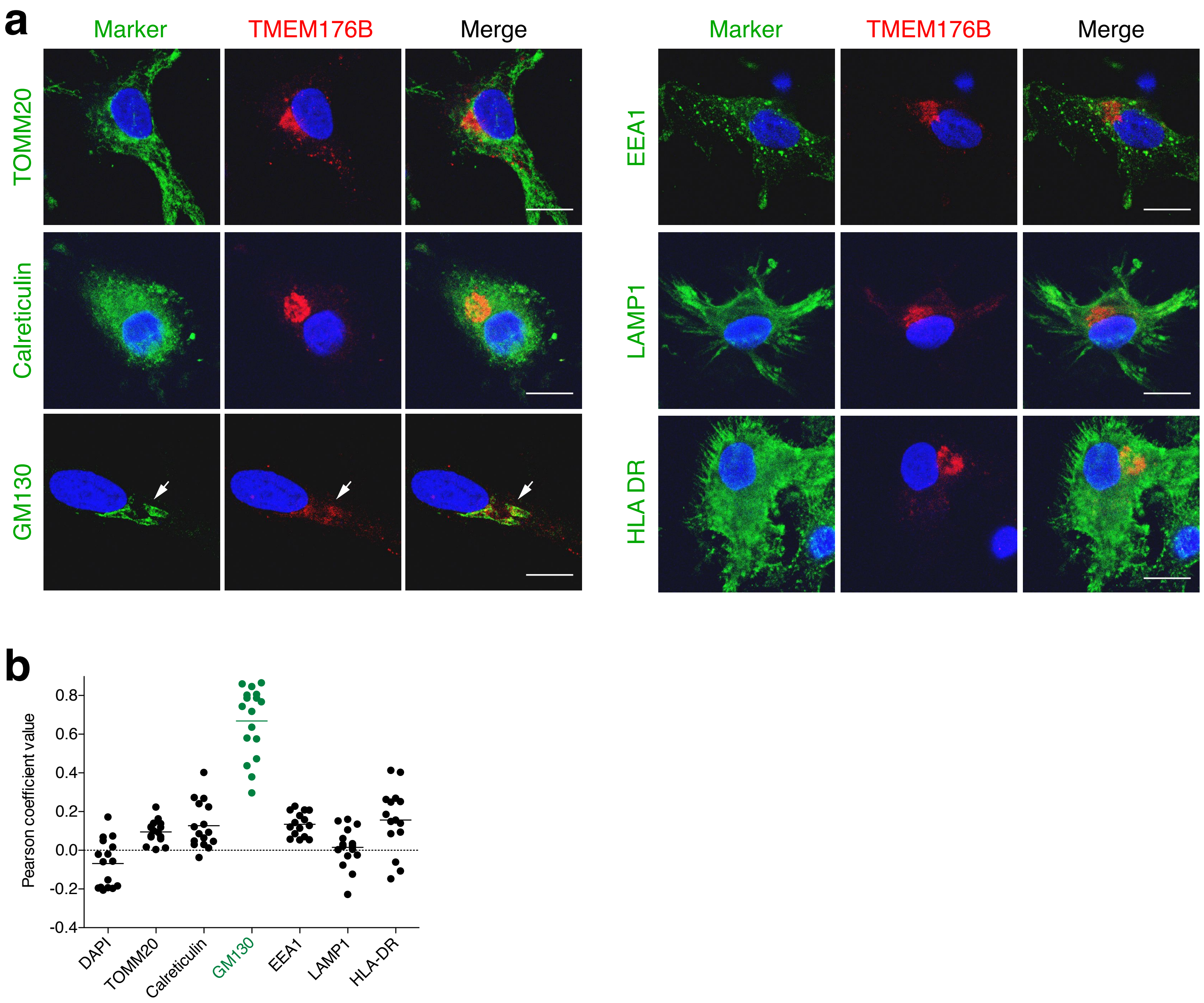
a
Marker
TMEM176B
Merge
TOMM20
Calreticulin
GM130
EEA1
LAMP1
HLA DR
b
Pearson coefficient value
0.8
0.6
0.4
0.2
0.0
-0.2
-0.4
DAPI
TOMM20
Calreticulin
GM130
EEA1
LAMP1
HLA-DR

Figure S4

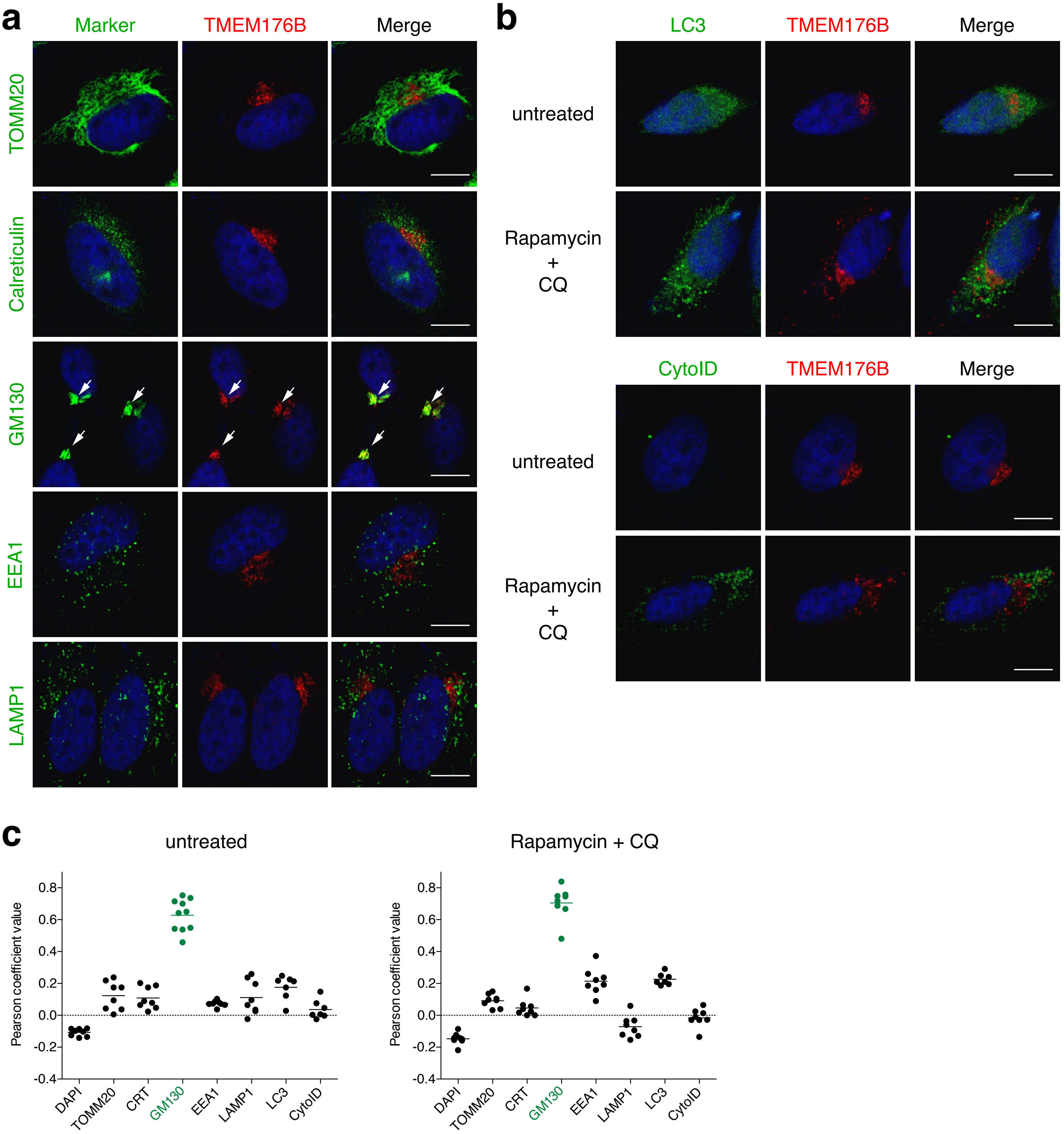
a
Marker
TMEM176B
Merge
TOMM20
Calreticulin
GM130
EEA1
LAMP1
b
LC3
TMEM176B
Merge
untreated
Rapamycin
+
CQ
CytoID
TMEM176B
Merge
untreated
Rapamycin
+
CQ
c
untreated
Rapamycin + CQ
Pearson coefficient value
0.8
0.6
0.4
0.2
0.0
-0.2
-0.4
DAPI
TOMM20
CRT
GM130
EEA1
LAMP1
LC3
CytoID

Supplementary Methods

Isolation of mouse intestinal lamina propria lymphocytes

Large and small intestines were harvested from adult mice (10-20 weeks of age). Fat tissues and Peyer's patches were removed. Intestines were flushed with PBS, cut open longitudinally and then cut into 1 cm pieces. Intestine pieces were incubated for 10 min at 37°C with horizontal shaking (100 rpm, 50-mL tube in a horizontal position) in 10 mL (for each mouse) of PBS containing 15 mM EDTA and 1.5 mM DTT, then vigorously shaken (using vortex for 45 sec and manually for 30 sec) and washed in PBS. The same cycle was repeated three times in PBS containing 15 mM EDTA (no DTT). After these steps aiming at removing eptihelial cells, intestine pieces were further processed into smaller fragments in a Petri dish with 5 mL of RPMI 1640 medium (Invitrogen) containing 2 mg/mL Collagenase D (Sigma). Five mL of Collagenase-medium supplemented with 200 µg/mL (then 100 µg/mL final in 10mL) DNase I (Sigma) were added and digestion was performed in a 50-mL tube for 30 min at 37°C with shaking. After suspension with a 10-mL pipet, the digested tissues were passed through a stainless steel mesh followed by successive filtrations through 100 µm and 40 µm cell strainers that were rinsed each time with 10 mL of PBS containing 2% fetal calf serum (FCS) and 2 mM EDTA (PFE) (~30 mL final). Undigested intestine pieces were subjected to a second cycle of digestion before pooling both cell preparations in 1 mL of PFE. Typical preparations yielded 10–30 x 10^6 live cells.

Electrophysiology

Whole-cell patch clamp was performed as previously described[3]. Briefly, *Xenopus laevis* female oocytes were then injected with 40 nL of in vitro synthesized (mMESSAGE mMACHINE Ultra

Kit) *Tmem176a* or/and *Tmem176b* mRNA at 1 mg/mL. The day after injection, the oocytes were placed in a pH 8 solution (in mM, NaCl, 100; KCl, 3; MgCl2, 2; HEPES, 15; pH 8) that was changed daily. Two to three days later, currents were recorded in two-electrode voltage-clamp using a genclamp500 amplifier (Axon Inst., Foster City, CA) interfaced to a personal computer using the Digidata 1200 interface and the pClamp software (ver 7.0; Axon Inst.). Prior to recording, oocytes were incubated in phorbol myristate acetate (PMA) at 100 nM in the pH 8 solution for 20–30 min. Currents were filtered at 100 Hz and digitized at 0.5 kHz before storage and further analysis. During recording, oocytes were continuously superfused with the pH 8 solution. On TMEM176A or B-expressing oocytes, induction of an inward current was obtained by switching to a pH 5 solution (in mM NaCl, 100; KCl, 3; MgCl2, 2; MES, 15; pH 5).

Supplemental Tables

Supplementary Table 1. Primers used for quantitative PCR.

Mouse gene name	Forward primer	Reverse primer	Amplicon (bp)
Foxp3	CCCAGGAAAGACAGCAACCTT	CTGCTTGGCAGTGCTTGAGAA	133
Ccl20	CTCACCTCTGCAGCCAGG	TCTGCTTTGGATCAGCGCAC	183
Cxcl3	CCCAGACAGAAGTCATAGCCAC	CCCAGACAGAAGTCATAGCCAC	193
Gapdh	GGTGAAGGTCGGTGTGAACGG	TCGCTCCTGGAAGATGGTGAT	232
Gata3	CCCATTACCACCTATCCGCC	GCATTGCAAAGGTAGTGCCC	209
Il17a	AGTCCAGGGAGAGCTTCATCT	TCTTCATTGCGGTGGAGAGTC	248
Il17f	GAAGTGCACCCGTGAAACAG	AACTGGAGCGGTTCTGGAAT	230
Il22	CCTACATGCAGGAGGTGGTG	AAACAGCAGGTCCAGTTCCC	176
Lcn2	ATGTCACCTCCATCCTGGTC	GCGAACTGGTTGTAGTCCGT	169
Ngp	CCACTCCGCCTTCTAGTCAG	AATCCCTGTCGCAGGTCAAG	198
Rorc	GCGCACCAACCTCTTTTCAC	AGGACGACTTCCATTGCTCC	201
Sgk1	ATGCAGTAAACCAAGCCGGT	CAGCAGGGCCAGTGTACTTC	209
Tbx21	GTCTGGGAAGCTGAGAGTCG	CTTTCCACACTGCACCCACT	229
Tmem176a	CAAACTTCTGCTGGCCGGAT	GTGAAGGAAGGCAACAGCTC	238
Tmem176b	AAGAAGTTTCTCTCCTGGCCT	CAGTTTTCCCTGCCTCTTCTCA	222

Human gene name	Forward primer	Reverse primer	Amplicon (bp)
IL17A	GACCTCATTGGTGTCACTGCT	CAAGGTGAGGTGGATCGGTT	207
RORC	CAGAGCCAAGGCTCAGTCAT	ATGGGGCAGTTCTGCTGAC	179
TBX21	ACAACCACCTGTTGTGGTCC	GACGTACAGGCGGTTTCCTG	239
TMEM176A	ACCTCCTTCATGGACATGCT	GCATGGCTAGATTCCACTCAC	183
TMEM176B	GCCATTGTCCATGAGAAGCA	ATTGCTGTGAACAACTTCCTCAG	281

Supplementary Table 2. Antibodies used for FACS analysis and sorting.

Mouse experiment	Antigen specificity	Fluorochrome	Clone	Manufacturer	Catalog #
$CD4^+$ Tconv and Treg isolation	CD45.2	APC-Cy7	104	BD Biosciences	560694
	CD4	PE-Cy7	RM4-5	BD Biosciences	552775
	Neuropilin-1	APC	761705	R&D Systems	FAB5994A
	TCRβ	PE	H57-597	BD Biosciences	562841
Naive $CD4^+$ T cell isolation for Th/iTreg in vitro polarization	CD4	V450	RM4-5	BD Biosciences	560468
	CD25	PE	7D4	BD Biosciences	558642
	CD44	PerCP-Cy5.5	IM7	BD Biosciences	560570
	CD62L	APC	MEL-14	BD Biosciences	561919
ILC3, ILC1 and $CD11b/c^+$ isolation	CD11b	biotin	M1/70	BD Biosciences	553309
	CD11c	biotin	HL3	BD Biosciences	553800
	CD19	APC	1D3	BD Biosciences	550992
	CD45.2	APC-Cy7	104	BD Biosciences	560694
	NK1.1	PE-Cy7	PK136	BD Biosciences	552878
	NKp46	FITC	29A1.4	BD Biosciences	560756
	TCRβ	APC	H57-597	eBioscience	17-5961-81
	TCRγδ	APC	Gl3	eBioscience	17-5711-82
	Streptavidin	PerCP-Cy5.5	-	BD Biosciences	551419
Naive $CD4^+CD45RB^{hi}$ T cells isolation for adoptive transfer into *Rag1*$^{-/-}$ mice	CD4	A647	RM4-5	BD Biosciences	557681
	CD25	PE-Cy7	PC61	BD Biosciences	552880
	CD45RB	FITC	16A	BD Biosciences	553100
$CD4^+TCR\beta^+$ and $T\gamma\delta^+$ T cell isolation from IMQ-treated mice	CD4	V450	RM4-5	BD Biosciences	560468
	TCRβ	APC	H57-597	eBioscience	17-5961-81
	TCRγδ	PE	Gl3	eBioscience	12-5711-82

Human experiment	Antigen specificity	Fluorochrome	Clone	Manufacturer	Catalog #
Naive $CD4^+$ T cell isolation for Th1/17 in vitro polarization	CD3	PE-Cy7	SK7	BD Biosciences	557851
	CD4	PerCP-Cy5.5	L200	BD Biosciences	552838
	CD45RA	PE	HI100	BD Biosciences	555489
	CD45RO	FITC	UCHL1	BD Biosciences	555492

Supplementary Table 3. Antibodies used for confocal analysis.

Primary antibodies (unconjugated except for LC3 : biotin)

Antigen specificity	Isotype	Clone	Manufacturer	Catalog #
Calreticulin	Mouse IgG1	16/Calreticulin	BD Biosciences	612136
CD3e	Mouse IgG2a, κ	HIT3a	BD Biosciences	555337
EEA1	Mouse IgG1	14/EEA1	BD Biosciences	610456
GM130	Mouse IgG1, κ	35/GM130	BD Biosciences	610822
HLA-DR	Mouse IgG2a, κ	G46-6	BD Biosciences	555810
LAMP1/CD107a	Mouse IgG1, κ	H4A3	BD Biosciences	555798
LC3	Mouse IgG1	5F10	Nanotools	0231-100BIOTIN/LC3-5F10
TGN46	Sheep	Polyclonal	AbD Serotec	AHP500GT
TMEM176A	Rat	Polyclonal	Biotem	-
TMEM176B	Rabbit	Polyclonal	Abcam	ab103929
TOMM20	Mouse IgG1, κ	4F3	Sigma	WH0009804M
HA	Mouse IgG3	HA.C5	Abcam	ab18181
V5	Mouse IgG2a	-	Life Technologies	R960-25

Secondary antibodies and streptavidin

Reactivity	Fluorochrome	Isotype	Manufacturer	Catalog #
Mouse IgG	Alexa Fluor 488	Goat	Life Technologies	A-11029
Mouse IgG1	Alexa Fluor 488	Goat	Life Technologies	A-21121
Mouse IgG2a	Alexa Fluor 568	Goat	Life Technologies	A-21134
Mouse IgG2a	Alexa Fluor 488	Goat	Life Technologies	A-21131
Mouse IgG3	Alexa Fluor 488	Goat	Life Technologies	A-21151
Rabbit IgG	Alexa Fluor 568	Goat	Life Technologies	A-11031
Rabbit IgG	Alexa Fluor 647	Goat	Life Technologies	A-21245
Rat IgG	Alexa Fluor 568	Goat	Life Technologies	A-11077
Sheep	biotin	Donkey	Jackson ImmunoResearch	713-066-147
Streptavidin	Alexa Fluor 488	-	Life Technologies	S11223
Streptavidin	Alexa Fluor 647	-	Life Technologies	S21374

Supplementary References

1 Omasits, U., Ahrens, C. H., Muller, S. & Wollscheid, B. Protter: interactive protein feature visualization and integration with experimental proteomic data. *Bioinformatics* **30**, 884-886, doi:10.1093/bioinformatics/btt607 (2014).

2 Mizushima, N., Yoshimori, T. & Levine, B. Methods in mammalian autophagy research. *Cell* **140**, 313-326, doi:10.1016/j.cell.2010.01.028 (2010).

3 Segovia, M. *et al.* Autologous dendritic cells prolong allograft survival through Tmem176b-dependent antigen cross-presentation. *Am J Transplant* **14**, 1021-1031, doi:10.1111/ajt.12708 (2014).
